# Research on the interaction between energy consumption and power battery life during electric vehicle acceleration

**DOI:** 10.1038/s41598-023-50419-3

**Published:** 2024-01-02

**Authors:** Qin Liu, Zhongbo Zhang, Jingjing Zhang

**Affiliations:** School of Mechatronic Engineering and Automation, Foshan University, Foshan, 528225 China

**Keywords:** Electrical and electronic engineering, Energy science and technology

## Abstract

Most studies on the acceleration process of electric vehicle focus on reducing energy consumption, but do not consider the impact of the power battery discharge current and its change rate on the battery life. Therefore, this paper studied the interaction between electric vehicle energy consumption and power battery capacity attenuation during acceleration. First, a power battery life model for electric vehicle under driving conditions is established, and the percentage of battery capacity loss per kilometer is used to measure the capacity loss under different acceleration conditions. Then, the relationship between the percentage of battery capacity loss per kilometer and velocity and acceleration is explored, and the capacity attenuation mechanism of power battery under different acceleration processes is analyzed. Finally, the energy consumption and battery capacity attenuation is studied when the electric vehicle accelerated with multiple accelerations curves, and the interaction of the first acceleration and acceleration time on the electric vehicle energy consumption and the power battery capacity attenuation characteristics is discussed. The research results indicate that when the electric vehicle accelerates with different multiple accelerations curves, the change of energy consumption per kilometer and percentage of battery capacity loss per kilometer with acceleration and acceleration time is different, and the change of the two is basically opposite.

## Introduction

The existing research on the electric vehicle (EV) energy consumption mainly focuses on the matching of powertrain parameters, the development of efficient control strategies for motor drive systems, and the optimization control strategies for energy management, and so on. For example, in the aspect of parameter matching, it mainly includes improving the working efficiency of each component of the powertrain system and reducing EV energy consumption, the parameters of the electric motor, battery and its transmission system are matched reasonably^1–4^. In the aspect of motor efficiency optimization control strategy, so as to reduce EV energy consumption and improve driving range, a series of motor efficiency optimization control strategies have been developed to achieve efficient operation of motors in high power range^5–7^. In the aspect of energy management optimization control strategy, fuzzy logic, dynamic planning and other control strategies are used to reduce electric vehicle energy consumption rate, thereby reducing EV energy consumption and increasing the driving range^8–11^.

Moreover, the research in references^12,13^ found that the magnitude of acceleration has a significant impact on EV energy consumption. However, there are few existing studies in EVs’ acceleration. For instance, in reference^14^, the difference of EV energy consumption under a single acceleration value and multiple accelerations values is studied. The research indicates that, compared to a single acceleration, when the EV accelerates with multiple accelerations values, its energy consumption is less. In reference^15^, the image of driving mode on the EV energy consumption is studied, and it is found that the energy consumption is lower when the EV is driven in a driving mode with a lower average velocity. Besides, the impact of different environmental and control parameters on energy consumption and driving range characteristics is studied in^16^; the research results indicate that the main factors affecting energy consumption and driving distance are average vehicle velocity, driving time, etc. Therefore, drivers can obtain more energy-efficient routes by estimating the driving distance under different driving conditions. And the relationship between EV energy consumption and acceleration time is discussed in^17^, and the results show that when the acceleration time is extended within an appropriate range, the energy consumption can be effectively reduced, and the lower the speed, the greater the energy saving potential.

And in terms of the analysis of power battery life, the main focus is on the prediction of power battery cycle life model. There are three prediction methods for power battery life: model-based prediction, data-driven prediction and fusion technology prediction^18–20^. Such as, an empirical model is used in reference^21^ to model the global and local degradation of lithium-ion battery aging process. In^22^, a physics model-based method is designed to predict battery capacity and remaining useful life, using a semi-empirical model, degradation parameters are estimated from voltage and capacity measurements to predict capacity decay trends. And in^23^, a new customary model is proposed to predict the battery degradation trend, the Bayesian inference technology is used to estimate the model parameters, particle filter step by step estimation and Markov chain Monte Carlo algorithm batch estimation, and then the estimated parameters are used to generate transient events and predict future capacity degradation. In addition, in reference^24^, a new degradation prediction model that integrates the effects of temperature and charge discharge cycles on battery degradation is established, and the PSO algorithm is applied to optimize the model parameters. And in reference^25^, the data-driven method based on discrete wavelet transform is proposed to prediction the remaining useful life of lithium-ion battery. Besides, Ahwiadi et al.^26^ used enhanced particle filter to improve the modeling accuracy of battery health monitoring and remaining useful life prediction. And in order to improve prediction accuracy, Duan et al.^27^ used the extended Kalman filter as sampling density function to optimize particle filter algorithm. The fusion method can be divided into two types: model and data-driven fusion method and different data-driven fusion method. For instance, Wei et al.^28^ used the fusion method of optimal correlation vector and improved degradation model with Hausdorff distance to predict the remaining useful life of lithium-ion battery. Wang et al.^29^ used the hybrid model of support vector regression and differential evolution to predict the remaining useful life of lithium ion battery. Zhu et al.^30^ used battery degradation data and Bayesian updating algorithm to establish the based-performance degradation Wiener process model. And in reference^31^, a hybrid method based on bidirectional long short-term memory model with attention mechanism and support vector regression model is proposed for online battery cycle life prediction.

On the basis of previous research, on the one hand, many studies are put into effect in reducing EV energy consumption by different control strategies, and the driving mode and influencing factors have also been analyzed. On the other hand, most research on power batteries mainly focuses on its remaining useful life. However, few studies have combined acceleration and acceleration time with EV energy consumption and battery life. Therefore, in this paper, the interaction mechanism between energy consumption and power battery life during the acceleration process of EVs is studied. The impact of vehicle velocity and acceleration on energy consumption and battery life is analyzed, considering the characteristic of the discharge rate of power batteries used in EVs constantly changing with the driving conditions, the capacity attenuation model of power battery under driving conditions is established, and the effect of discharge current and its change on battery capacity attenuation is discussed. In addition, when the EV accelerates with convex acceleration curves with multiple accelerations values, the interaction relationship between energy consumption and power battery capacity attenuation is also studied, and the variation of energy consumption and battery life with acceleration and acceleration time is analyzed. The research in this paper can provide guidance for the optimization of the EV acceleration mode, so as to further reduce the energy consumption and thus prolong the battery life.

## Mathematical model of EV energy consumption and battery life

### The model of EV energy consumption during accelerating process

According to the principle of longitudinal dynamics of automobile^32^, when the EV accelerates on good road, the output power of its power battery $$P_{{\text{b}}} (t)$$$$\left( {\text{W}} \right)$$ at time *t* can be obtained as:

1 $$P_{{\text{b}}} (t) = \frac{{F_{{\text{t}}} (t) \cdot u\left( t \right)}}{{3.6\eta_{{\text{T}}} \eta_{{\text{V}}} \eta_{{\text{m}}} }}$$

where the $F_{{\text{t}}} (t)$ (N) is traction force; $\eta_{T}$ is the efficiency of the transmission system; $\eta_{{\text{V}}}$ is the efficiency of the inverter; $\eta_{{\text{m}}}$ is the efficiency of the electric motor.

Therefore, when an EV accelerates according to different acceleration conditions, the energy consumption per kilometer $E_{{{\text{b}} - {\text{j}}}}^{l} \left( t \right)$ in the $l$ acceleration range $\left[ {t_{l} \sim t_{l + 1} } \right]$ is:

2 $$E_{{{\text{b}} - {\text{j}}}}^{l} \left( t \right) = \frac{{\int_{{t_{l} }}^{{t_{l + 1} }} {P_{{\text{b}}} (t)dt} }}{{\int_{{t_{l} }}^{{t_{l + 1} }} {u(t)dt} }}$$

By bringing the traction force into the above formula, the EV energy consumption per kilometer of the $l$ acceleration range $\left[ {t_{l} \sim t_{l + 1} } \right]$ can be obtained as follows:

3 $$E_{{{\text{b}} - {\text{j}}}}^{l} \left[ {u\left( t \right),a\left( t \right)} \right]{ = }\frac{{\int_{{t_{l} }}^{{t_{l + 1} }} {{{u\left( t \right) \cdot \left[ {mgf + mgi + \frac{{C_{{\text{D}}} Au\left( t \right)^{2} }}{21.15} + \delta ma\left( t \right)} \right]} \mathord{\left/ {\vphantom {{u\left( t \right) \cdot \left[ {mgf + mgi + \frac{{C_{{\text{D}}} Au\left( t \right)^{2} }}{21.15} + \delta ma\left( t \right)} \right]} {3.6\eta_{{\text{T}}} \eta_{{\text{V}}} \eta_{{\text{m}}} }}} \right. \kern-0pt} {3.6\eta_{{\text{T}}} \eta_{{\text{V}}} \eta_{{\text{m}}} }}dt} }}{{\int_{{t_{l} }}^{{t_{l + 1} }} {u\left( t \right)dt} }}$$

The above equation can also be expressed as:

4 $$E_{{{\text{b}} - {\text{j}}}}^{l} \left[ {u\left( t \right),a\left( t \right)} \right]{ = }\frac{{\int_{{t_{l} }}^{{t_{l + 1} }} {\left[ {k_{{\text{f}}} u\left( t \right) + k_{{{\text{C}}_{{\text{D}}} }} u^{3} \left( t \right) + k_{{\updelta }} a\left( t \right) \cdot u\left( t \right)} \right]dt} }}{{\int_{{t_{l} }}^{{t_{l + 1} }} {u\left( t \right)dt} }}$$

In Eq. (4), $k_{{\text{f}}}$ is defined as the impact factor of road resistance, $k_{{\text{f}}} = {{\left( {mgf + mgi} \right)} \mathord{\left/ {\vphantom {{\left( {mgf + mgi} \right)} {3.6\eta_{{\text{T}}} \eta_{{\text{V}}} \eta_{{\text{m}}} }}} \right. \kern-0pt} {3.6\eta_{{\text{T}}} \eta_{{\text{V}}} \eta_{{\text{m}}} }}$; $k_{{{\text{C}}_{{\text{D}}} }}$ is defined as the impact factor of air resistance, $k_{{{\text{C}}_{{\text{D}}} }} = {{C_{D} A} \mathord{\left/ {\vphantom {{C_{D} A} {76.14 \cdot \eta_{{\text{T}}} \eta_{{\text{V}}} \eta_{{\text{m}}} }}} \right. \kern-0pt} {76.14 \cdot \eta_{{\text{T}}} \eta_{{\text{V}}} \eta_{{\text{m}}} }}$; $k_{{\updelta }}$ is defined as the impact factor of acceleration resistance, $k_{{\updelta }} = {{\delta m} \mathord{\left/ {\vphantom {{\delta m} {3.6\eta_{{\text{T}}} \eta_{{\text{V}}} \eta_{{\text{m}}} }}} \right. \kern-0pt} {3.6\eta_{{\text{T}}} \eta_{{\text{V}}} \eta_{{\text{m}}} }}$.

Then, the EV energy consumption per kilometer $E_{{\text{b - j}}} \left[ {u\left( t \right),a\left( t \right)} \right]$ during the whole acceleration process can be expressed as:

5 $$E_{{{\text{b}} - {\text{j}}}} \left[ {u\left( t \right),a\left( t \right)} \right]{ = }\frac{{\sum\nolimits_{l = 1}^{n} {\int_{{t_{l} }}^{{t_{l + 1} }} {\left[ {k_{{\text{f}}} u\left( t \right) + k_{{{\text{C}}_{{\text{D}}} }} u^{3} \left( t \right) + k_{{\updelta }} a\left( t \right) \cdot u\left( t \right)} \right]dt} } }}{{\sum\nolimits_{l = 1}^{n} {\int_{{t_{l} }}^{{t_{l + 1} }} {u\left( t \right)dt} } }}$$

From Eqs. (4) and (5), it can be concluded that during the acceleration process of an EV, when the vehicle parameters and road conditions are constant, its $k_{{\text{f}}}$, $k_{{{\text{C}}_{{\text{D}}} }}$ and $k_{{\updelta }}$ can be regarded as fixed values. The $E_{{{\text{b}} - {\text{j}}}} \left[ {u\left( t \right),a\left( t \right)} \right]$ mainly depends on $u\left( t \right)$ and $a\left( t \right)$, i.e. $E_{{{\text{b}} - {\text{j}}}} \left[ {u\left( t \right),a\left( t \right)} \right] = f\left( {u\left( t \right),a\left( t \right)} \right)$, while the $u\left( t \right)$ and $a\left( t \right)$ mainly depend on different acceleration conditions. Therefore, studying different acceleration processes and implementing reasonable control is an important way to reduce the EV energy consumption.

### The model of battery life during accelerating process

During the EV acceleration process, the power battery life mainly depends on the discharge rate $n$, temperature $T$, discharge depth $DOD$, etc. Among domestic and foreign scholars' research on the life model of lithium-ion batteries, the model established by Wang John^33^ of the American HRL laboratory is the most representative. In the study of the impact of $T$, $n$, and $DOD$ on battery capacity, the battery capacity loss rate was used to predict the battery life, and according to the experimental results in reference^33^, the battery capacity loss model of lithium-ion power batteries can be expressed as:

6 $$Q_{{{\text{loss}}}} = \left( {36042 - 9419n{ + }1215n^{2} - 48n^{3} } \right) \cdot \exp \left( {\frac{ - 31700 + 370.3 \times n}{{RT}}} \right) \times \left( {A_{{\text{h}}} } \right)^{0.55}$$

where $B$ is pre-exponential factor; $A_{h}$ (${\text{A}}\;{\text{h}}$) represents the cumulative ampere-hours, so it can be expressed as $A_{{\text{h}}} = \int_{0}^{t} {\left| {I\left( t \right)} \right|dt}$, $R$ is gas constant, $R = 8.314\;{\text{J/}}\left( {{\text{mol}}\;{\text{K}}} \right)$.

**Figure 1:**
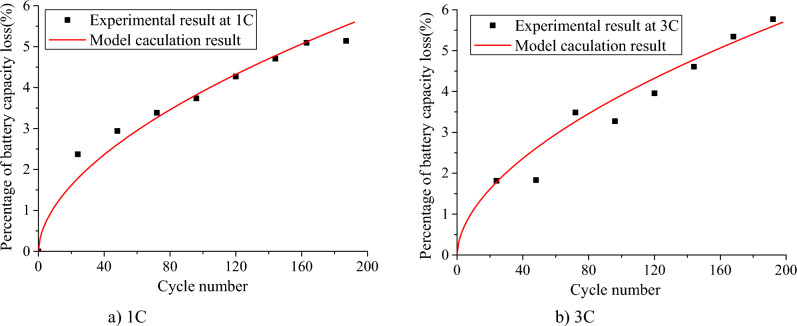
Comparison of theoretical calculation and experimental results of power battery capacity loss.

The Eq. (6) reflects the battery capacity loss of a single battery under constant current discharge conditions. To verify the correctness of the model and its applicability, a certain lithium-ion power battery for electric vehicle is used for charge–discharge experiments. Set the termination voltage of charge and discharge to 3.65 V and 2.5 V, and discharge at 1C and 3C, respectively. The test results are shown in Fig. 1.

As can be seen from Fig. 1, the percentage of battery capacity loss calculated by the model is basically consistent with the experimental results, and the experimental results of the percentage of battery capacity loss under 1C and 3C discharge are evenly distributed on both sides of the theoretical value. The model of formula (6) is only applicable to the life prediction analysis of a single battery under constant discharge rate. However, the working conditions of EVs are variable in the actual operation process, and the discharge current of its power battery varies greatly, and the corresponding discharge rate is not a constant value and fluctuates greatly. Therefore, it is necessary to revise the capacity loss model under constant discharge conditions, and refer to the method in reference^34^ to predict the battery life under actual driving conditions. Based on equal life loss, assuming that the battery capacity loss rate under 1C discharge rate is $Q_{{{\text{loss1}}}}$, and the battery capacity loss rate at nC discharge rate is $Q_{{{\text{loss}}n}}$, the battery capacity loss under nC discharge condition can be deduced from $Q_{{{\text{loss1}}}} = Q_{{{\text{loss}}n}}$ as follows:

7 $$Q_{{{\text{loss}}n{1}}} = B_{1} \cdot \exp \left( {\frac{ - 31329.7}{{RT}}} \right) \times \left\{ {\left[ {\frac{{B_{n} }}{{B_{1} }} \cdot \exp \left( {\frac{370.3 \cdot n - 370.3}{{RT}}} \right)} \right]^{{\frac{1}{0.55}}} \cdot A_{{{\text{h}}n}} } \right\}^{0.55}$$

where $$B_{1} = 27790$$, $$B_{n} { = }36042 - 9419n{ + }1215n^{2} - 48n^{3}$$。

Since the acceleration process is studied in this paper, only the discharge process of the power battery is considered, then the capacity attenuation $Q_{{\text{loss - j}}}^{l} \left( t \right)$ of the power battery in the $l$ acceleration range $\left[ {t_{l} \sim t_{l + 1} } \right]$ can be expressed as:

8 $$Q_{{\text{loss - j}}}^{l} \left( t \right) = B_{1} \cdot \exp \left( {\frac{ - 31329.7}{{RT}}} \right) \times \left\{ {\int_{{t_{l} }}^{{t_{l + !} }} {\left[ {\frac{{B_{n} }}{{B_{1} }} \cdot \exp \left( {\frac{370.3 \cdot n\left( t \right) - 370.3}{{RT}}} \right)} \right]^{{\frac{1}{0.55}}} \cdot \frac{{n(t) \times I_{1} }}{3600}dt} } \right\}^{0.55}$$

where $I_{1}$ is the current at 1C discharge rate of the single battery; $n\left( t \right)$ is the discharge rate at time *t*, $n\left( t \right) = {{I\left( t \right)} \mathord{\left/ {\vphantom {{I\left( t \right)} {C_{{\text{r}}} }}} \right. \kern-0pt} {C_{{\text{r}}} }}$, $C_{{\text{r}}}$ is the nominal capacity of a single battery, and $I\left( t \right)$ is the discharge current of a single battery during accelerating driving. Ignoring the impact of single battery inconsistency, assuming that the current flowing through each parallel module is consistent, the current of the single battery is $I\left( t \right) = {{I_{{{\text{pack}}}} \left( t \right)} \mathord{\left/ {\vphantom {{I_{{{\text{pack}}}} \left( t \right)} {Num}}} \right. \kern-0pt} {Num}}$, and $Num$ is the parallel number of the battery pack. The current of the battery pack is $I_{{{\text{pack}}}} \left( t \right) = I_{{\text{b}}} \left( t \right)$, and the output current of the power battery pack $I_{{\text{b}}} \left( t \right)$ can be expressed as $I_{{\text{b}}} \left( t \right) = {{P_{{\text{b}}} (t)} \mathord{\left/ {\vphantom {{P_{{\text{b}}} (t)} {U_{{\text{b}}} \left( t \right)}}} \right. \kern-0pt} {U_{{\text{b}}} \left( t \right)}}$. The voltage fluctuation of the power battery pack during the EV acceleration process is also very small, which can be regarded as a constant. Therefore, the capacity attenuation of the power battery in an acceleration condition can be obtained by substituting the corresponding parameters:

9 $$Q_{{\text{loss - j}}}^{l} \left[ {u\left( t \right),a\left( t \right)} \right] = B_{1} \cdot \exp \left( {\frac{ - 31329.7}{{RT}}} \right) \times \left\{ {\int_{{t_{l} }}^{{t_{l + 1} }} \begin{gathered} \left[ {\frac{{B_{n} }}{{B_{1} }} \cdot \exp \left( {\frac{370.3 \cdot n\left( t \right) - 370.3}{{RT}}} \right)} \right]^{{\frac{1}{0.55}}} \cdot \hfill \\ \frac{{\left[ {k_{{\text{f}}} u\left( t \right) + k_{{{\text{C}}_{{\text{D}}} }} u^{3} \left( t \right) + k_{{\updelta }} a\left( t \right) \cdot u\left( t \right)} \right]}}{3600 \cdot U \cdot Num}dt \hfill \\ \end{gathered} } \right\}^{0.55}$$

The percentage of battery capacity loss per kilometer $q_{{{\text{loss}} - {\text{j}}}}^{l} \left[ {u\left( t \right),a\left( t \right)} \right]\left( {{\text{\% /km}}} \right)$ in an acceleration condition can be expressed as:

10 $$q_{{{\text{loss}} - {\text{j}}}}^{l} \left[ {u\left( t \right),a\left( t \right)} \right]{ = }\frac{{Q_{{{\text{loss}} - {\text{j}}}}^{l} \left[ {u\left( t \right),a\left( t \right)} \right]}}{{\int_{{t_{l} }}^{{t_{l + 1} }} {u\left( t \right)dt} }}$$

And the $q_{{{\text{loss}} - {\text{j}}}} \left[ {u\left( t \right),a\left( t \right)} \right]$ of the power battery of an EV during the entire acceleration process can be expressed as:

11 $$q_{{{\text{loss}} - {\text{j}}}} \left[ {u\left( t \right),a\left( t \right)} \right]{ = }\frac{{\sum\nolimits_{l = 1}^{n} {Q_{{{\text{loss}} - {\text{j}}}}^{l} \left[ {u\left( t \right),a\left( t \right)} \right]} }}{{\sum\nolimits_{l = 1}^{n} {\int_{{t_{l} }}^{{t_{l + 1} }} {u\left( t \right)dt} } }}$$

From the above analysis, it can be concluded that during the EV acceleration process, when the vehicle parameters and road conditions are constant, the $k_{{\text{f}}}, k_{{{\text{C}}_{{\text{D}}} }}$ and $k_{{\updelta }}$ can also be considered as fixed values. Then the $q_{{{\text{loss}} - {\text{j}}}} \left[ {u\left( t \right),a\left( t \right)} \right]$ mainly depends on $u\left( t \right)$ and $a\left( t \right)$, that is $q_{{{\text{loss}} - {\text{j}}}} = f\left[ {u\left( t \right),a\left( t \right)} \right]$, and $u\left( t \right)$ and $a\left( t \right)$ mainly depend on the acceleration curve. Therefore, studying the different acceleration processes of EVs and implementing reasonable control is also a way to extend the battery life. And Table 1 shows the main parameters of the EV and the battery.

**Table 1:** The main parameters of EV and battery.

Parameter	Value	Parameter	Value
Vehicle mass, *m* (kg)	2295	Nominal voltage of single cell (V)	3.2
Drag coefficient*,C*_*D*_	0.28	Nominal capacity of single cell, $C_{r}$ (Ah)	10
Frontal area, *A* (m^2^)	2.67	The number cells in parallel,$Num$	18
Rolling friction coefficient, *f*	0.01	Total battery voltage, $U$ (V)	316.8
Vehicle rotating mass conversion factor, *δ*	1.04	Total battery capacity (Ah)	180

## Research on the relationship between EV energy consumption and power battery Life with multiple acceleration curves

On the basis of research in reference^35^, for a given acceleration condition which the initial velocity and the final velocity and the total acceleration time are constant, when the EV accelerates with convex multiple accelerations curves, its energy consumption per kilometer is lower than using a single acceleration. Therefore, this section analyzes the influence of the number of acceleration and the acceleration time in the multiple accelerations curves on the EV energy consumption and the battery life under different acceleration conditions. Select three acceleration conditions with zero initial velocity and final velocity of 40 km/h, 80 km/h, and 120 km/h respectively to analyze the impact of different acceleration numbers on energy consumption per kilometer and percentage of battery capacity loss per kilometer. Among them, 0–40 km/h is defined as the neighborhood condition, 0–80 km/h is defined as the urban condition, and 0–120 km/h is defined as the highway condition. Taking into account the actual acceleration conditions, the number of accelerations cannot be infinitely large, refer to the limits of the number of accelerations in^14^, in this paper, the value of acceleration number *n* is $n \le 6$. The acceleration time and the acceleration for three working conditions are set as shown in Table 2.

**Table 2:** The setting of different acceleration conditions.

Acceleration condition (km/h)	Acceleration range (m/s^2^)	Acceleration time range (s)	Acceleration time setting (s)	The number of acceleration
0–40	[0.1 3.0]	[3.7 111]	10–30	$\le 6$
0–80	[0.1 3.0]	[7.4 222]	10–60	$\le 6$
0–120	[0.1 3.0]	[11.1 333]	10–90	$\le 6$

**Figure 2:**
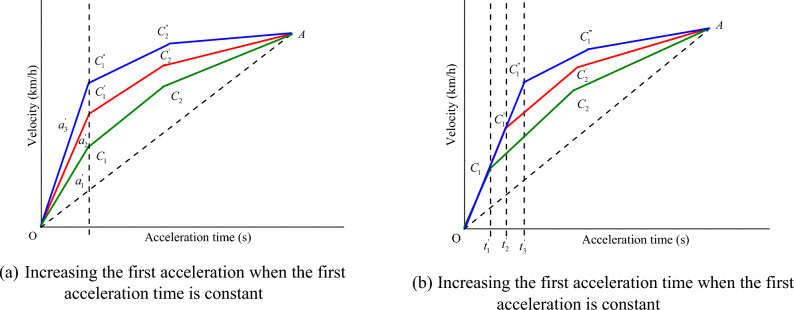
The vehicle velocity change with three acceleration values.

According to the setting of acceleration time and acceleration under different working conditions in the table, combined with the previous research^33^, this article mainly delves into the changes of $$E_{{\text{b - j}}} \left[ {u\left( t \right),a\left( t \right)} \right]$$ and $$q_{{{\text{loss}} - {\text{j}}}} \left[ {u\left( t \right),a\left( t \right)} \right]$$ with acceleration and acceleration time, when the EV accelerates with three acceleration values. Figure 2 shows the relationship between vehicle velocity and acceleration time, let the acceleration and acceleration time of the first segment be $$a_{1}^{{{\text{III}}}}$$ ($${\text{m/s}}^{{2}}$$) and $$t_{{1}}^{{{\text{III}}}}$$ (s) respectively, the acceleration and acceleration time of the second segment be $$a_{{2}}^{{{\text{III}}}}$$ ($${\text{m/s}}^{{2}}$$) and $$t_{{2}}^{{{\text{III}}}}$$ (s) respectively, the acceleration and acceleration time of the third segment be $$a_{{3}}^{{{\text{III}}}}$$ ($${\text{m/s}}^{{2}}$$) and $$t_{{3}}^{{{\text{III}}}}$$ (s) respectively, and $$a_{1}^{{{\text{III}}}} > a_{{2}}^{{{\text{III}}}} > a_{{3}}^{{{\text{III}}}}$$, and the final acceleration velocity and acceleration time be $$u_{{\text{f}}}^{{{\text{III}}}}$$ ($${\text{km/h}}$$) and $$t_{{\text{f}}}^{{{\text{III}}}}$$ (s) respectively. Assuming that $$a_{1}^{{{\text{III}}}} ,t_{1}^{{{\text{III}}}}$$ is known, then $$a_{{3}}^{{{\text{III}}}}$$ and $$t_{{3}}^{{{\text{III}}}}$$ can be expressed by $$u_{{\text{f}}}^{{{\text{III}}}} ,t_{{\text{f}}}^{{{\text{III}}}} ,a_{1}^{{{\text{III}}}} ,t_{1}^{{{\text{III}}}} ,a_{2}^{{{\text{III}}}} ,t_{2}^{{{\text{III}}}}$$. Therefore, when the EV accelerates with a three acceleration curve, the variation of its energy consumption per kilometer $$E_{{{\text{b}} - {\text{j}}}}^{{{\text{III}}}}$$ $$\left( {\text{Wh/km}} \right)$$ and the percentage of battery capacity loss per kilometer $$q_{{{\text{loss}}}}^{{{\text{III}}}}$$$$\left( {{\text{\% /km}}} \right)$$ with $$a_{2}^{{{\text{III}}}}$$ and $$t_{2}^{{{\text{III}}}}$$ can be analyzed. Considering two situations in this section, the first is to increase $$a_{1}^{{{\text{III}}}}$$ when $$t_{1}^{{{\text{III}}}}$$ is constant, and analyze the variation of the $$E_{{\text{b - j}}}^{{{\text{III}}}}$$ and the $$q_{{{\text{loss}}}}^{{{\text{III}}}}$$ with $$a_{2}^{{{\text{III}}}}$$ and $$t_{2}^{{{\text{III}}}}$$, as shown in Fig. 2a, where $$a_{1}{\prime} < a_{2}{\prime} < a_{3}{\prime}$$, the second is to increase $$t_{1}^{{{\text{III}}}}$$ when $$a_{1}^{{{\text{III}}}}$$ is constant, and analyze the variation of the $$E_{{\text{b - j}}}^{{{\text{III}}}}$$ and the $$q_{{{\text{loss}}}}^{{{\text{III}}}}$$ with $$a_{2}^{{{\text{III}}}}$$ and $$t_{2}^{{{\text{III}}}}$$, as shown in Fig. 2b, where $$t_{1}{\prime} < t_{2}{\prime} < t_{3}{\prime}$$.

### Increasing the first acceleration

**Figure 3:**
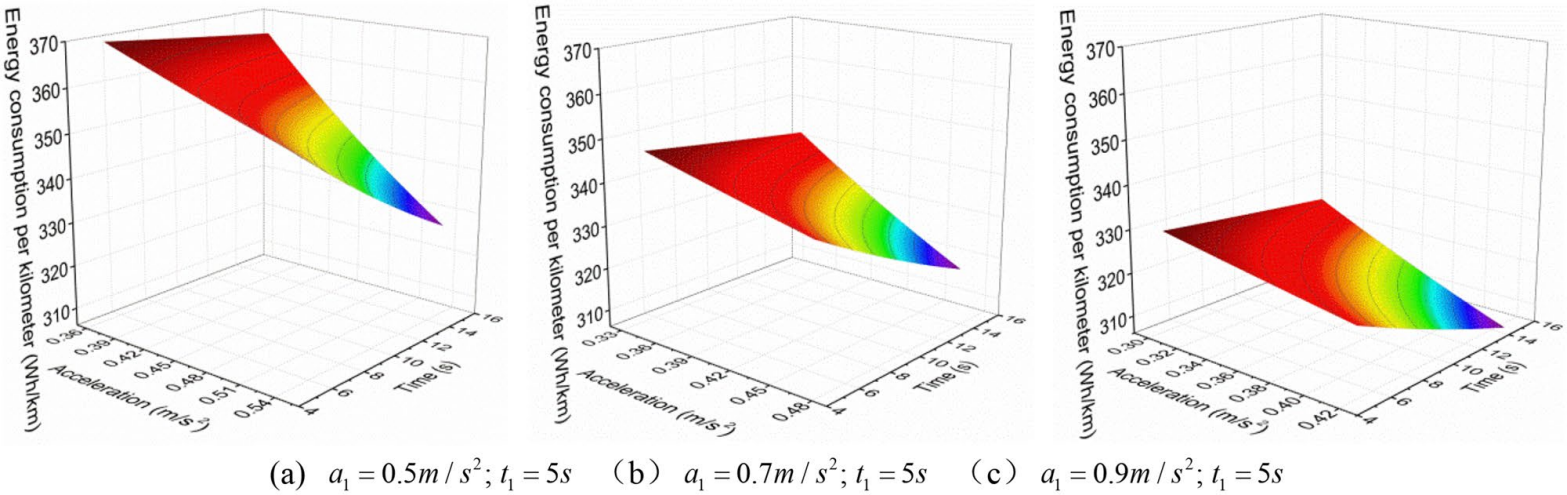
The $E_{{\text{b - j}}}^{{{\text{III}}}}$ change under neighborhood condition (0–40 km/h) with three acceleration values.

**Figure 4:**
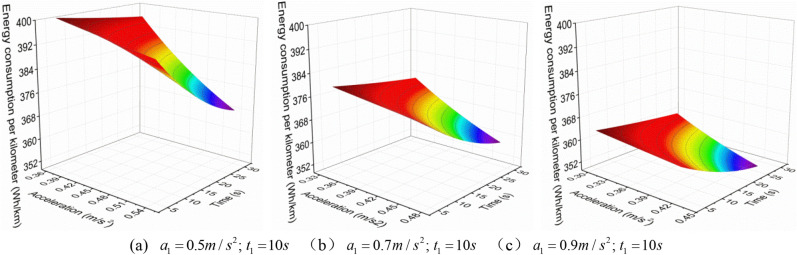
The $E_{{\text{b - j}}}^{{{\text{III}}}}$ change under urban condition (0–80 km/h) with three acceleration values.

**Figure 5:**
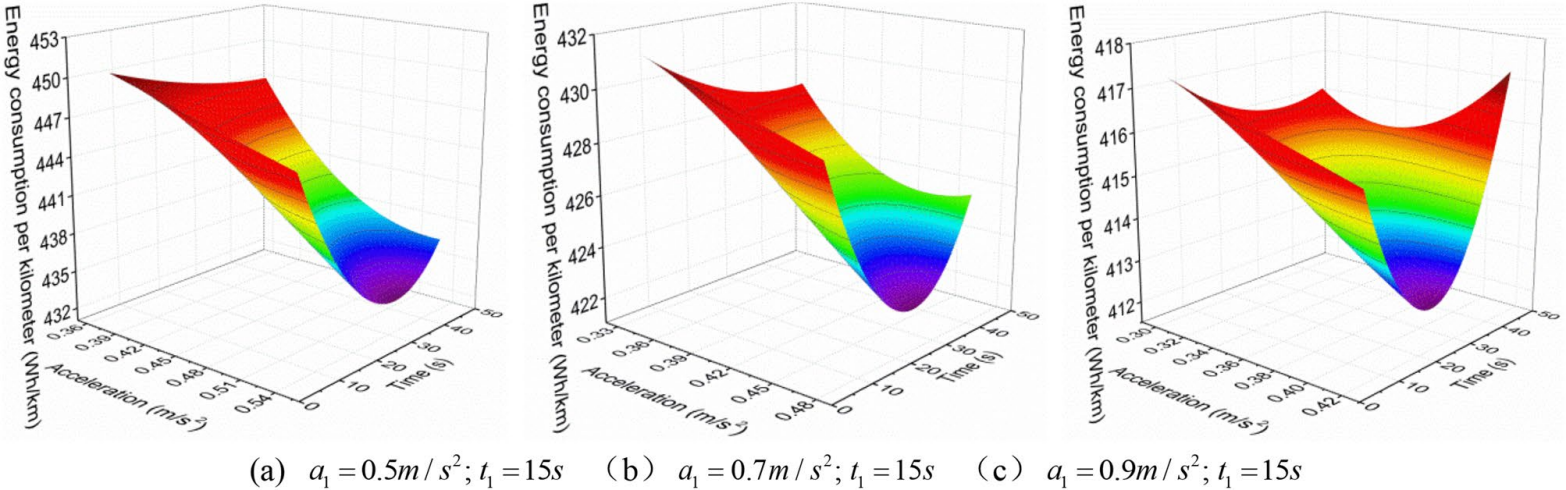
The $E_{{\text{b - j}}}^{{{\text{III}}}}$ change under highway condition (0–120 km/h) with three acceleration values.

Firstly, analyze the first scenario, which is when the EV accelerates with three accelerations, the first acceleration $a_{1}^{{{\text{III}}}}$ is increased when the first acceleration time $t_{1}^{{{\text{III}}}}$ is constant, and the variation of the $E_{{\text{b - j}}}^{{{\text{III}}}}$ with the $a_{2}^{{{\text{III}}}}$ and the $t_{2}^{{{\text{III}}}}$ is shown in Figs. 3, 4 and 5.

**Figure 6:**
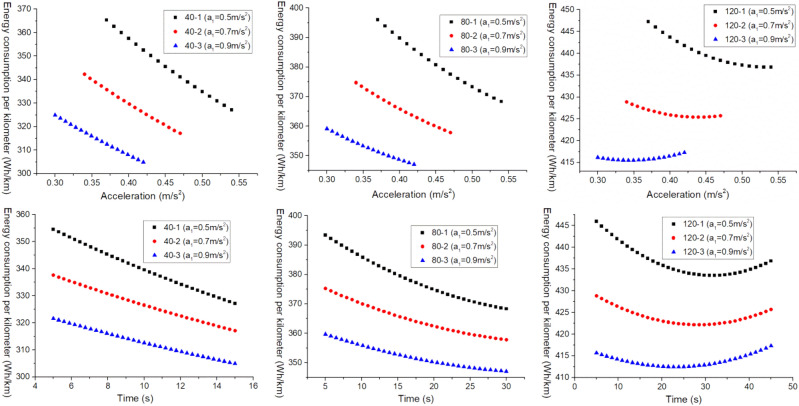
The $E_{{\text{b - j}}}^{{{\text{III}}}}$ change under different working conditions with three acceleration values.

For the purpose of further analyzing the relationship between the $E_{{\text{b - j}}}^{{{\text{III}}}}$ and the $a_{2}^{{{\text{III}}}}$ and the $t_{2}^{{{\text{III}}}}$, and for comparative analysis, a certain boundary is intercepted from Figs. 3, 4 and 5 for analysis, as shown in Fig. 6.

As shown in Figs. 3, 4 and 5, when the EV accelerates with three acceleration values, for the neighborhood condition, the $E_{{{\text{b}} - {\text{j}}}}^{{{\text{III}}}}$ gradually decreases as the increase of $a_{2}^{{{\text{III}}}}$ and $t_{2}^{{{\text{III}}}}$. However, for the urban condition and highway condition, the $E_{{{\text{b}} - {\text{j}}}}^{{{\text{III}}}}$ decreases first and then increases as the increase of $a_{2}^{{{\text{III}}}}$ and $t_{2}^{{{\text{III}}}}$, and the higher the velocity, the greater the $E_{{{\text{b}} - {\text{j}}}}^{{{\text{III}}}}$, and the more obvious this change trend. This indicates that under a given operating condition, when the EV accelerates with three acceleration curve, and $a_{1}^{{{\text{III}}}} ,t_{1}^{{{\text{III}}}}$ are certain, there is an optimal point ($a_{2}^{{{\text{III}}}}$, $t_{2}^{{{\text{III}}}}$) where $E_{{{\text{b}} - {\text{j}}}}^{{{\text{III}}}}$ is minimized.

In addition, from Fig. 6, it can be observed that under the same working condition, when the $t_{1}^{{{\text{III}}}}$ remains constant, the $E_{{{\text{b}} - {\text{j}}}}^{{{\text{III}}}}$ decreases as the $a_{1}^{{{\text{III}}}}$ increases. This indicates that when the EV accelerates with three acceleration values, and when the $t_{1}^{{{\text{III}}}}$ is constant, the bigger the $a_{1}^{{{\text{III}}}}$, the smaller the $E_{{{\text{b}} - {\text{j}}}}^{{{\text{III}}}}$. Therefore, when the EV accelerates with multiple acceleration curves, the EV energy consumption can be reduced by appropriately increasing the first segment acceleration.

**Figure 7:**
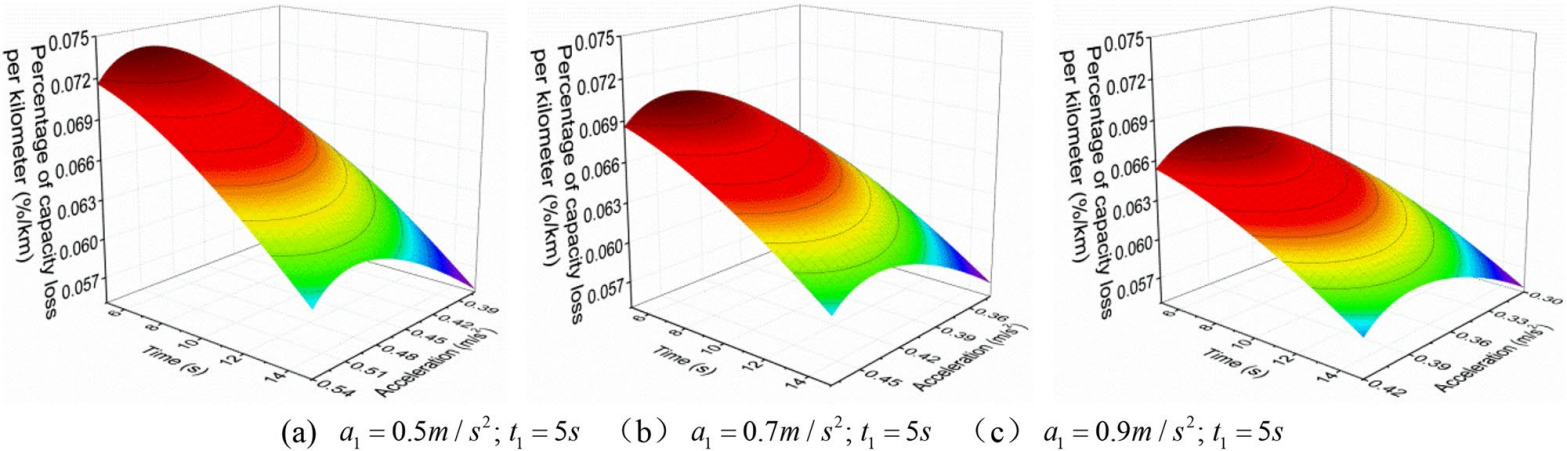
The $q_{{{\text{loss}}}}^{{{\text{III}}}}$ change under neighborhood condition (0–40 km/h) with three acceleration values.

**Figure 8:**
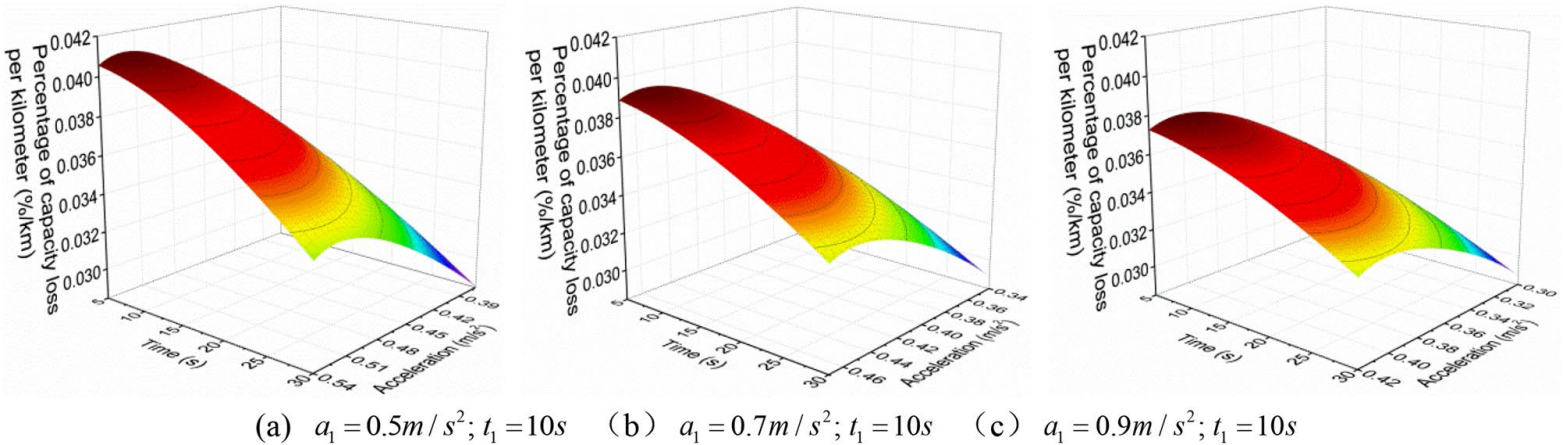
The $q_{{{\text{loss}}}}^{{{\text{III}}}}$ change under urban condition (0–80 km/h) with three acceleration values.

**Figure 9:**
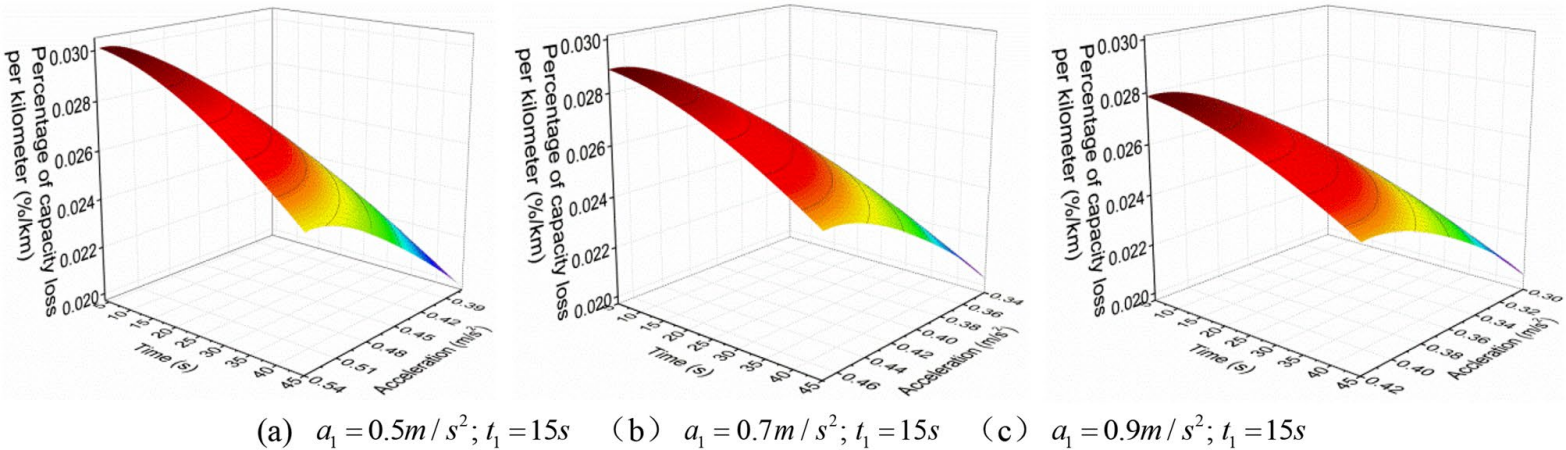
The $q_{{{\text{loss}}}}^{{{\text{III}}}}$ change under highway condition (0–120 km/h) with three acceleration values.

When the EV accelerates with a convex acceleration curve of three accelerations values, the $a_{1}^{{{\text{III}}}}$ is increased when the $t_{1}^{{{\text{III}}}}$ is constant, the relationship between $q_{{{\text{loss}}}}^{{{\text{III}}}}$ and $a_{2}^{{{\text{III}}}}$ and $t_{2}^{{{\text{III}}}}$ is shown in Figs. 7, 8 and 9.

**Figure 10:**
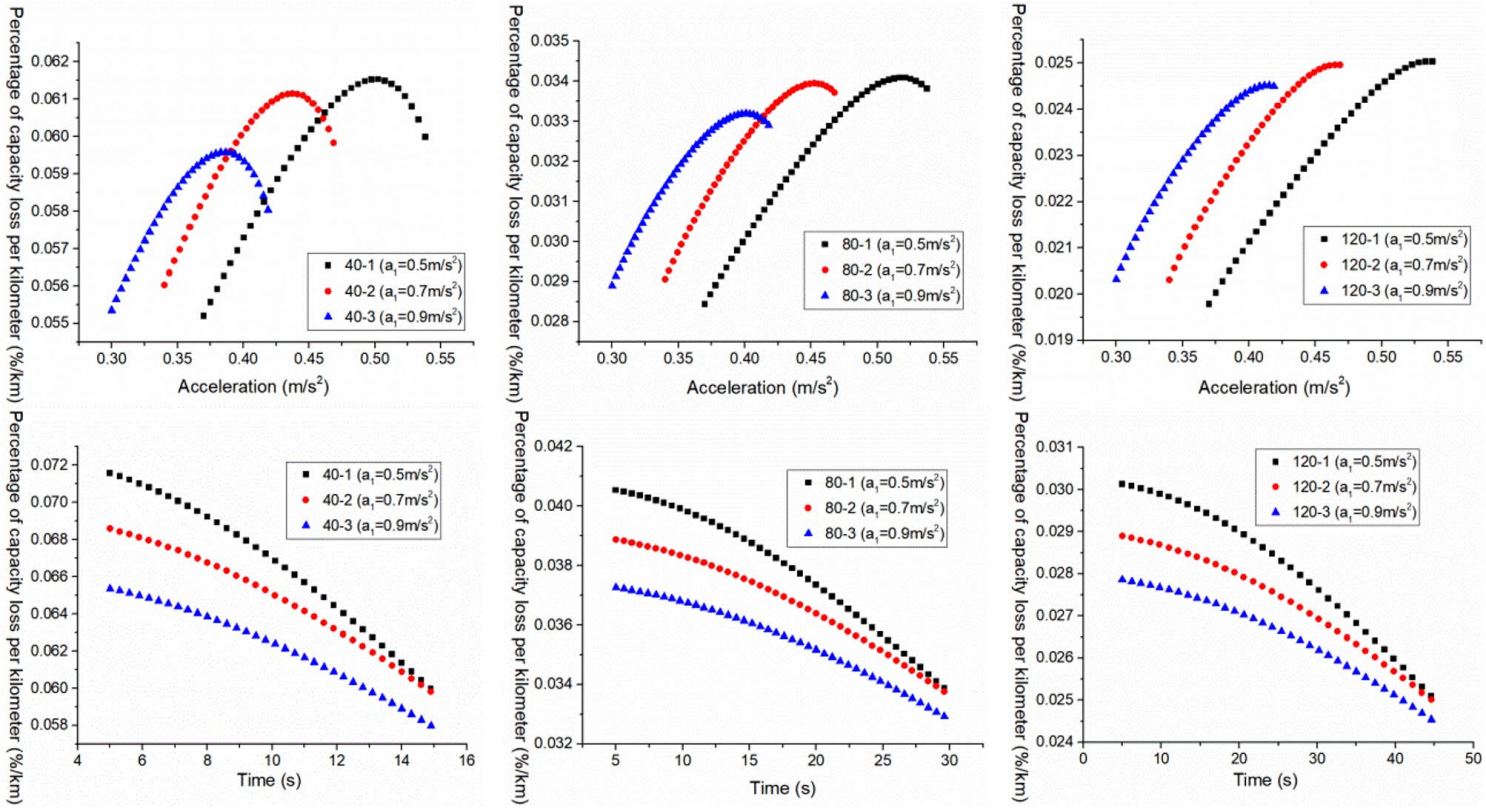
The $q_{{{\text{loss}}}}^{{{\text{III}}}}$ change under different working conditions with three acceleration values.

Similarly, in order to more intuitively analyze the relationship between $q_{{{\text{loss}}}}^{{{\text{III}}}}$ and $a_{2}^{{{\text{III}}}}$ and $t_{2}^{{{\text{III}}}}$ under different working conditions, the boundary data in Figs. 7, 8 and 9 are intercepted to analyze, as shown in Fig. 10.

From Figs. 7, 8 and 9, it can be found that when the EV accelerates with the convex curve of three accelerations values, under three acceleration conditions of neighborhood, urban, and highway, its $q_{{{\text{loss}}}}^{{{\text{III}}}}$ gradually decreases with the increase of the $t_{2}^{{{\text{III}}}}$, and the $q_{{{\text{loss}}}}^{{{\text{III}}}}$ increases first and then decreases with the increase of the $a_{2}^{{{\text{III}}}}$. Moreover, the lower the vehicle velocity, the more obvious this change trend is, and the higher the vehicle velocity, the smaller the $q_{{{\text{loss}}}}^{{{\text{III}}}}$.

In addition, from Fig. 10, it can also be found that under the same acceleration condition, when the $t_{1}^{{{\text{III}}}}$ remains constant and increases the $a_{1}^{{{\text{III}}}}$, the variation of $q_{{{\text{loss}}}}^{{{\text{III}}}}$ is different. As shown in the figure, under the same $a_{2}^{{{\text{III}}}}$, the larger $a_{1}^{{{\text{III}}}}$, the greater $q_{{{\text{loss}}}}^{{{\text{III}}}}$; And under the same $t_{2}^{{{\text{III}}}}$, the larger $a_{1}^{{{\text{III}}}}$, the smaller $q_{{{\text{loss}}}}^{{{\text{III}}}}$, which is different from the variation trend of the $E_{{\text{b - j}}}^{{{\text{III}}}}$.with $a_{2}^{{{\text{III}}}}$ and $t_{2}^{{{\text{III}}}}$.

### Increasing the first acceleration time

And then analyze the second scenario, that is, when the EV accelerates with a convex acceleration curve of three accelerations values, the $t_{1}^{{{\text{III}}}}$ is increased when the $a_{1}^{{{\text{III}}}}$ is constant, and the variation of $E_{{\text{b - j}}}^{{{\text{III}}}}$ and $q_{{{\text{loss}}}}^{{{\text{III}}}}$ with $a_{2}^{{{\text{III}}}}$ and $t_{2}^{{{\text{III}}}}$ is analyzed. Based on the analysis in “Increasing the first acceleration”, in order to further analyze the relationship between $E_{{\text{b - j}}}^{{{\text{III}}}}$ and $q_{{{\text{loss}}}}^{{{\text{III}}}}$ and $a_{2}^{{{\text{III}}}}$ and $t_{2}^{{{\text{III}}}}$, as well as compare and analyze the changes in $E_{{\text{b - j}}}^{{{\text{III}}}}$ and $q_{{{\text{loss}}}}^{{{\text{III}}}}$ under different acceleration conditions, this section directly intercepts its boundary data for analysis.

**Figure 11:**
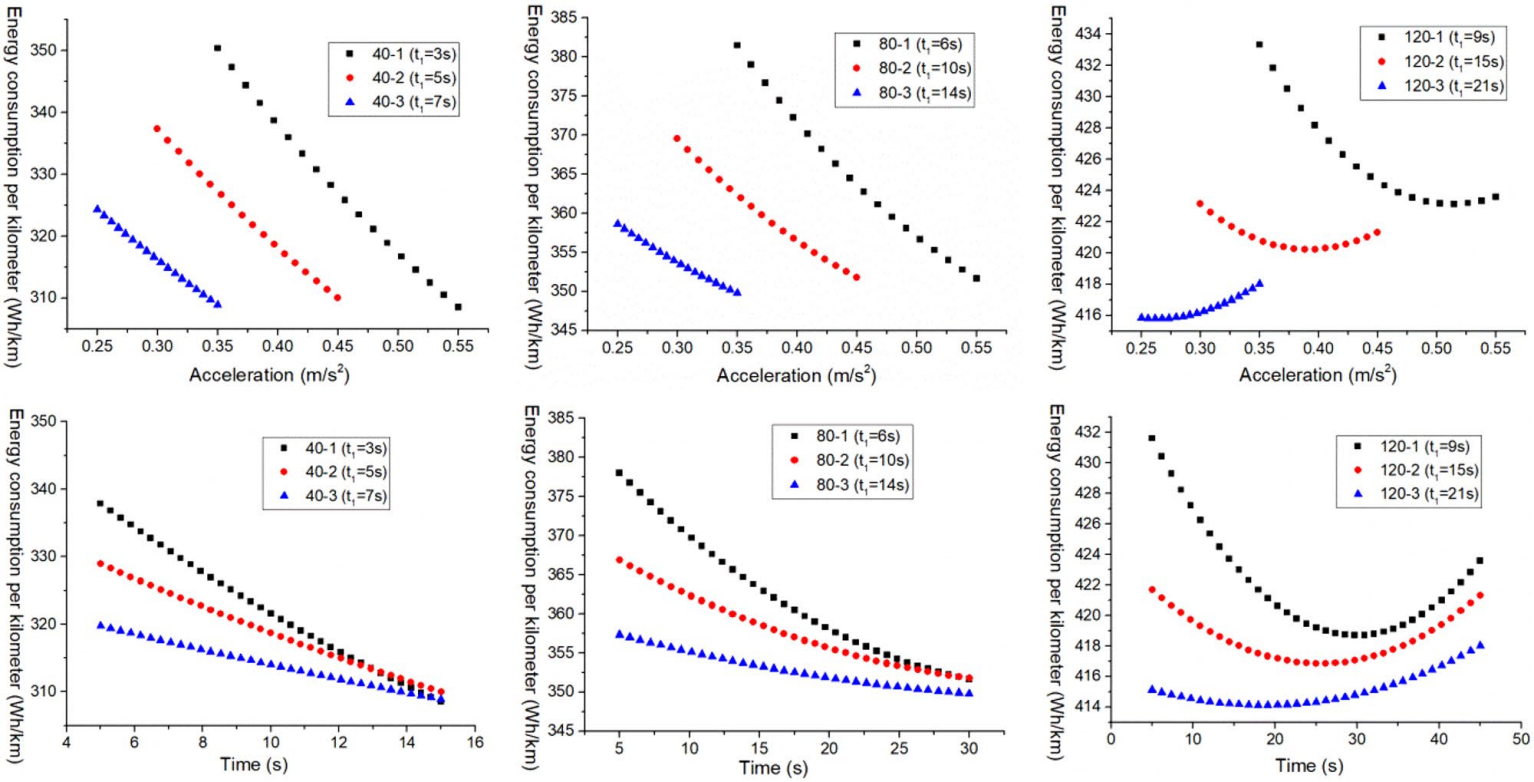
The $E_{{\text{b - j}}}^{{{\text{III}}}}$ change under different working conditions with three acceleration values.

As shown in Fig. 11, the variation of $E_{{{\text{b}} - {\text{j}}}}^{{{\text{III}}}}$ with $a_{2}^{{{\text{III}}}}$ and $t_{2}^{{{\text{III}}}}$ under different acceleration conditions are shown.

From the overall variation trend in Fig. 11, it can be seen that under neighborhood conditions, the $E_{{{\text{b}} - {\text{j}}}}^{{{\text{III}}}}$ decreases with the increase of $a_{2}^{{{\text{III}}}}$ and $t_{2}^{{{\text{III}}}}$. However, under urban and highway conditions, the $E_{{{\text{b}} - {\text{j}}}}^{{{\text{III}}}}$ shows a trend of first decreasing and then increasing with the increase of $a_{2}^{{{\text{III}}}}$ and $t_{2}^{{{\text{III}}}}$, and the higher the vehicle velocity, the more obvious the $E_{{{\text{b}} - {\text{j}}}}^{{{\text{III}}}}$ change. This indicates that under a given acceleration condition, when an EV accelerates with a three acceleration curve and $a_{1}^{{{\text{III}}}} ,t_{1}^{{{\text{III}}}}$ is constant, there is an optimal $a_{2}^{{{\text{III}}}} ,t_{2}^{{{\text{III}}}}$ that where the $E_{{{\text{b}} - {\text{j}}}}^{{{\text{III}}}}$ is minimized.

Besides, it can also be observed that under the same acceleration condition, when $a_{1}^{{{\text{III}}}}$ remains unchanged, the $E_{{{\text{b}} - {\text{j}}}}^{{{\text{III}}}}$ shows a decreasing trend with the increase of $t_{1}^{{{\text{III}}}}$. This indicates that when the electric vehicle accelerates with three accelerations, moreover, the $a_{1}^{{{\text{III}}}}$ is constant, the longer the $t_{1}^{{{\text{III}}}}$, the smaller the $E_{{{\text{b}} - {\text{j}}}}^{{{\text{III}}}}$. Therefore, when the EV accelerates with multiple acceleration curves, its energy consumption can be reduced by appropriately extending the acceleration time of the first segment.

**Figure 12:**
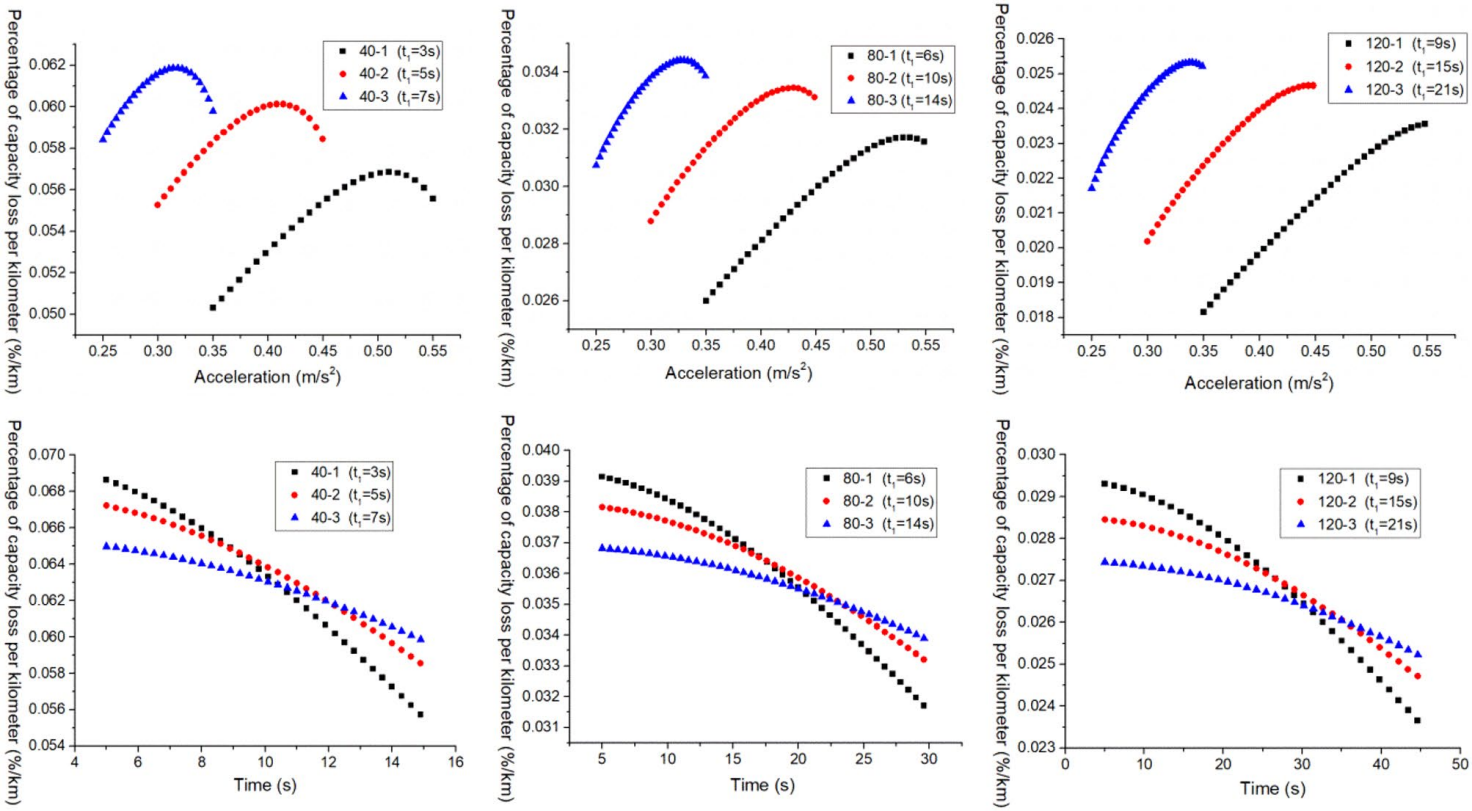
The $q_{{{\text{loss}}}}^{{{\text{III}}}}$ change under different working conditions with three acceleration values.

And the relationship between the $q_{{{\text{loss}}}}^{{{\text{III}}}}$ and $a_{2}^{{{\text{III}}}}$ and $t_{2}^{{{\text{III}}}}$ is shown in Fig. 12.

As can be seen from Fig. 12, when the EV accelerates with a convex acceleration curve of three accelerations values, under three acceleration conditions of neighborhood, urban and highway, its $q_{{{\text{loss}}}}^{{{\text{III}}}}$ decreases with the increase of $t_{2}^{{{\text{III}}}}$, and presents a trend of first increasing and then decreasing with the increase of $a_{2}^{{{\text{III}}}}$. The lower the vehicle velocity, the more obvious the change trend is, and the higher the vehicle velocity, the smaller the $q_{{{\text{loss}}}}^{{{\text{III}}}}$. In addition, it can also be seen that under the same acceleration condition, when the $a_{1}^{{{\text{III}}}}$ remains unchanged, the $q_{{{\text{loss}}}}^{{{\text{III}}}}$ increases as the $t_{1}^{{{\text{III}}}}$ increases, which is exactly contrary to the variation trend of the $E_{{{\text{b}} - {\text{j}}}}^{{{\text{III}}}}$.

In summary, when the EV accelerates with different multiple acceleration curves, the changes in energy consumption per kilometer and percentage of battery capacity loss per kilometer with acceleration and acceleration time are different, and the changes in the two are basically opposite. For instance, when the EV accelerates with a three acceleration curve, increasing $a_{1}^{{{\text{III}}}}$ and $t_{1}^{{{\text{III}}}}$ appropriately may help reduce $E_{{{\text{b}} - {\text{j}}}}^{{{\text{III}}}}$, but $q_{{{\text{loss}}}}^{{{\text{III}}}}$ will increase. Therefore, the two are contradictory, so in the study of the acceleration process of electric vehicles, it is not only considered energy consumption, but also the impact of the magnitude and number of accelerations in the multiple acceleration curves on the power battery capacity attenuation characteristics.

## Conclusion

When the EV accelerates with different acceleration curves, its energy consumption and battery life are different. In this paper, the interaction mechanism between the EV energy consumption and the battery capacity loss under different multiple accelerations curves is studied, and when the EV accelerates with neighborhood, urban, highway conditions, the relationship between the energy consumption, battery capacity attenuation, acceleration and acceleration time is analyzed. The research results indicate that when the EV accelerates with different multiple accelerations curves, their energy consumption and the battery capacity attenuation have different relationships with acceleration and acceleration time, and their variation patterns are basically opposite. Under neighborhood conditions, the $E_{{{\text{b}} - {\text{j}}}}^{{{\text{III}}}}$ decreases with the increase of $a_{2}^{{{\text{III}}}}$ and $t_{2}^{{{\text{III}}}}$; and under urban and highway conditions, the $E_{{{\text{b}} - {\text{j}}}}^{{{\text{III}}}}$ shows a trend of first decreasing and then increasing with the increase of $a_{2}^{{{\text{III}}}}$ and $t_{2}^{{{\text{III}}}}$, and the higher the vehicle velocity, the greater the $E_{{{\text{b}} - {\text{j}}}}^{{{\text{III}}}}$, and the more obvious this change trend. However, under three acceleration conditions, the $q_{{{\text{loss}}}}^{{{\text{III}}}}$ decreases with the increase of $t_{2}^{{{\text{III}}}}$, and presents a trend of first increasing and then decreasing with the increase of $a_{2}^{{{\text{III}}}}$. The lower the vehicle velocity, the more obvious this change trend, and the higher the vehicle velocity, the smaller the $q_{{{\text{loss}}}}^{{{\text{III}}}}$. Thus it can be seen that when the EV accelerates with a three accelerations curve, increasing the first acceleration and the first acceleration time appropriately can help reduce the energy consumption per kilometer, but the percentage of battery capacity loss per kilometer will increase. Therefore, the two are contradictory, so in the subsequent optimization of the acceleration process, not only energy consumption should be considered, but also the impact of the acceleration magnitude, the number of acceleration and acceleration time during acceleration process on the power battery life.

## List of symbols

$$F_{{\text{t}}} (t)$$: Traction force (N)
$u\left( t \right)$: EV velocity (km/h)
$\eta_{T}$: Efficiency of the transmission system
$\eta_{V}$: Efficiency of the inverter
$\eta_{{\text{m}}}$: Efficiency of the electric motor
$E_{{\text{b - j}}}^{l} \left( t \right)$: EV energy consumption per kilometer in the $l$ acceleration (Wh/km)
$$a\left( t \right)$$: Acceleration (m/s^2^)
$Q_{loss}$: Percentage of battery capacity loss (%)
B: Pre-exponential factor
$A_{h}$: Cumulative ampere-hours (${\text{A}}\;{\text{h}}$)
R: Gas constant ($${\text{J/}}\left( {{\text{mol}}\;{\text{K}}} \right)$$)
$Q_{{\text{loss - j}}}^{l} \left( t \right)$: Percentage of battery capacity loss in the $l$ acceleration (%)
$q_{{\text{loss - j}}}^{l} \left[ {u\left( t \right),a\left( t \right)} \right]$: Percentage of battery capacity loss per kilometer (%/km)
